# Manic Episode Induced by Lamotrigine in Rapid Cycling Bipolar Disorder

**DOI:** 10.7759/cureus.16184

**Published:** 2021-07-05

**Authors:** Ana Lúcia Ramos, Henrique Salgado, Miguel Bragança

**Affiliations:** 1 Psychiatry, University Hospital Center of Sao Joao, Oporto, PRT

**Keywords:** bipolar disorder, rapid cycling, lamotrigine, bipolar depression, mania

## Abstract

Bipolar disorder is characterized by persistent and/or recurrent mood changes between depressive and manic poles. Rapid cycling is a frequent, although underrecognized, condition in bipolar disorder, and it is known to worsen the prognosis of the disease. With regard to the treatment of bipolar depression, there is a shortage of evidence‐supported treatment choices, and the literature on the subject includes few references to cases of manic switch induced by lamotrigine.

The authors describe a case of a rapid cycling bipolar patient who presented manic symptoms after initiating treatment with lamotrigine.

## Introduction

Bipolar disorder is a chronic psychiatric disorder characterized by persistent and/or recurrent mood changes between depressive and manic poles [1]. The course of the disease is complex and heterogeneous, with a highly variable frequency of mood and energy transitions. About one in six patients who seek treatment for bipolar disorder present with a rapid-cycling pattern [2]. Rapid-cycling bipolar disorder refers to the presence of at least food mood episodes in the previous 12 months that meet the criteria for manic, hypomanic, or major depressive episodes [3]. Rapid-cycling bipolar disorder is associated with poorer treatment response, worse long-term prognosis, and probable higher suicide risk than bipolar disorder without rapid cycling [4]. Regarding the response to pharmacological measures, treatment resistance for each episode appears to increase with each additional recurrence [5]. Hypothyroidism, antidepressant use, and substance abuse are often associated with rapid cycling [6].

The treatment of bipolar disorder comprehends different strategies, which include the maintenance treatment and the approach of acute mania or depression. There are multiple factors that make the treatment of acute depression the most challenging step [7] and, in addition, there is a paucity of evidence‐supported treatment choices for bipolar depression [8]. The risk of manic switch is commonly feared, especially concerning rapid-cycling patients.

Lamotrigine is an effective treatment for acute bipolar depression [9], and it has a lower risk of adverse effects (although potentially severe rash) compared with alternatives as lithium and quetiapine [8]. Furthermore, in two large 18‐month trials, lamotrigine did not increase the risk of mania compared with placebo [8]. On the other hand, there are some described cases of manic episodes that occurred after the introduction of lamotrigine [9-12]. Therefore, although unlikely, we must be aware of this risk in clinical practice.

In this case report, we describe the clinical features of a patient with rapid cycling bipolar disorder that suffered a manic switch after lamotrigine introduction.

## Case presentation

A 59-year-old man, diagnosed with bipolar disorder I for many years, presented with an episode of bipolar depression with symptoms such as anhedonia, depressed mood, reduced energy, psychomotor retardation, demotivation, anxiety, and decreased social activity, which had been gradually worsening for two weeks. Significant impairment in his usual functioning was noted, given that the patient lost the interest and ability to work in the projects that excited him the most, for example, he abandoned the writing of a new book (months before, when stable, he managed to translate a book he had already published). He also stopped taking care of his diet, with less and less elaborate meals. These symptoms were interpreted as an episode of bipolar depression. At that time, he was being treated at the day hospital of the psychiatric department and was medicated with semisodium valproate 1500 mg id, olanzapine 10 mg 2id, aripiprazole 10 mg id, and lorazepam 2.5 mg 3id.

About six months before this episode, the patient was hospitalized for a period of six weeks following a suicide attempt through self-induced deep cuts in the upper limbs, with severe bleeding that motivated hospitalization in the intensive care unit. This event occurred in the context of severe depression with psychotic characteristics (with delusional ideas of guilt and ruin). In that time, medication was adjusted with the introduction of fluoxetine 20 mg id, olanzapine 10 mg id, and diazepam 5 mg 2id (in addition to semisodium valproate and lorazepam, which he was already taking). Initially, a clinical improvement was noticed, but, soon after discharge, he had a subsequent manic switch, with elevated mood, increased energy, psychomotor agitation, verborrea, disinhibition, involvement in new projects, and regular cocaine consumption. Thus, the medication was progressively readjusted and cessation of cocaine consumption was promoted, with progressive clinical improvement, until he turned to the depressive mood described at the beginning of this case report.

In addition to this recent psychiatric history, the patient had already been hospitalized six times in psychiatry wards due to decompensation of his bipolar disorder. In some of these hospitalizations, he also presented with mental and behavioral disorders induced by psychoactive substance use, which normally takes place after the installation of elevated mood and disinhibition. In fact, he has a history of drug abuse, mainly cocaine but also cannabinoids, heroin, and alcohol. His medical history also includes hypertension, treated with lisinopril 10 mg id and amlodipine 5 mg id.

Facing the current depressive episode, lab tests were carried out and no abnormalities were found in blood cell count, renal function, electrolytes, liver function, folic acid, B12 vitamin, total cholesterol, and glucose. Valproate levels were 61.7 mg/L and urine drug screening was negative. Regarding the treatment, it must be taken into account that, in previous depressive episodes, the patient responded poorly to quetiapine and reported serious adverse effects of lithium, such as muscle twitch and trembling, pointing out his low tolerability to the drug. Given this background, the decision was to start lamotrigine in titration up to 25 mg id, in addition to the semisodium valproate he was already taking. We also decided to suspend aripiprazole.

About three to four weeks after initiating lamotrigine, the patient presented with an expansive mood, sudden involvement in new projects (intention of publishing new books), increased energy, easy social interactions, disinhibition, fast speech, and insomnia. At that time, the dose of lamotrigine was 25 mg id, and the taking of the medication was supervised by the nurses in the day hospital. The patient himself early recognized that his mood was unwell, explaining his clinical condition with the introduction of lamotrigine: "lamotrigine is like methamphetamine, I am no longer depressed." After the onset of this manic episode, the patient resumed cocaine use and, at this point, we must highlight that the consumption only began after the exacerbation of manic symptoms, given that the previous drug screenings, regularly performed, were consistently negative. Therefore, lamotrigine was gradually discontinued, aripiprazole 10 mg was reintroduced, sodium valproate dose was increased to 2 g id, and flurazepam 30mg id was started. At the same time, cocaine use was discouraged again, and the patient stopped using it. About two weeks after the discontinuation of lamotrigine, a gradual clinical stabilization was observed, with a return to euthymic mood and normal speech debt, stable sleep and appetite, and clear absence of psychotic activity or suicidal ideation, overlapping his previous functioning.

## Discussion

This case report presents a patient who had two depressive episodes and two manic episodes in the period of one year, making him suitable for the diagnosis of rapid cycling bipolar disorder I. Rapid cycling is a frequent, although underrecognized, condition in bipolar disorder, and it contributes to a worse prognosis [3]. The literature suggests that rapid cycling affects a significant portion of bipolar patients and is related to a longer course of illness, earlier age at onset, more illegal drug and alcohol abuse, and increased suicidality [3], clinical features that were accessed in this patient. 

Despite the variety of clinical manifestations exhibited by the patient, it was clearly noticed that the biggest damage to his way of acting was caused by depressive episodes, as proven by the history of serious suicide attempts. Indeed, in bipolar disorder, symptoms of depression have been shown to account for more impairment than symptoms of mania [13]. Thus, early treatment of the acute depressive episode, as well as prevention of relapses, was of supreme importance in this case. As the patient had a history of manic switches induced by antidepressants, as well as poor response to quetiapine and low tolerance to lithium, we opted for the introduction of lamotrigine in his therapeutic scheme. In fact, in a previous study, lamotrigine was generally effective and well-tolerated in a group of previously non-responsive rapid cycling bipolar patients [14].

Although there is sparse evidence about the potential risk of a manic switch related to lamotrigine therapy, in this case, the manic symptoms were distinctly observed after the patient initiated lamotrigine. At the time, the interference of illicit psychoactive drugs in the symptoms was suspected, given the patient's history of substance abuse. However, urinary drug screenings were performed every two days, and since the results were consistently negative, the possibility that the patient was using drugs was ruled out. At the onset of the manic episode, the drug screening was negative for cocaine, opiates, cannabinoids, and amphetamines. By the way, it was interesting to note that the patient himself had a perception of the potential risk of a manic switch caused by lamotrigine, an understanding that was evident when he compared taking lamotrigine with being under the effect of stimulating drugs, such as amphetamines, in order to justify his elevated mood and energy.

Since we started to taper off lamotrigine soon after the first manic symptoms, it is questionable if a sufficient dose of lamotrigine was reached. However, the possibility of an insufficient dose is less likely, as the patient was taking semisodium valproate at the same time, which is known to raise the blood concentration of lamotrigine. On Naranjo Adverse Drug Reaction Probability Scale, the total score for lamotrigine was 3, which means that the symptoms were a possible adverse reaction to lamotrigine [15]. Nevertheless, we cannot rule out the possible contribution of aripiprazole withdrawal to the beginning of the manic symptoms.

Finally, despite the possible association between lamotrigine and the occurrence of the reported manic switch, we cannot exclude that this abrupt mood transition may have occurred in the context of the natural course of the disease, especially considering the rapid-cycling pattern and the history of drug use relapses.

## Conclusions

Even though the episodes of manic switch are not consistently linked to the therapy with lamotrigine, the number of cases described in the literature is not insignificant. In this case report, we describe a patient who presented with depressive symptoms and turned manic after the introduction of lamotrigine. This article aims to draw the attention of the scientific community to this possibility, highlighting the need for future studies on the subject.
